# The SpRY Cas9 variant release the PAM sequence constraint for genome editing in the model plant *Physcomitrium patens*

**DOI:** 10.1007/s11248-024-00381-1

**Published:** 2024-04-04

**Authors:** Julie Calbry, Guillaume Goudounet, Florence Charlot, Anouchka Guyon-Debast, Pierre-François Perroud, Fabien Nogué

**Affiliations:** Institut Jean-Pierre Bourgin (IJPB), Université Paris-Saclay, INRAE, AgroParisTech, 78000 Versailles, France

**Keywords:** CRISPR, PAMless Cas9, *Physcomitrium patens*, Gene editing, Off-target

## Abstract

Genome editing via CRISPR/Cas has enabled targeted genetic modifications in various species, including plants. The requirement for specific protospacer-adjacent motifs (PAMs) near the target gene, as seen with Cas nucleases like SpCas9, limits its application. PAMless SpCas9 variants, designed with a relaxed PAM requirement, have widened targeting options. However, these so-call PAMless SpCas9 still show variation of editing efficiency depending on the PAM and their efficiency lags behind the native SpCas9. Here we assess the potential of a PAMless SpCas9 variant for genome editing in the model plant *Physcomitrium patens*. For this purpose, we developed a SpRYCas9i variant, where expression was optimized, and tested its editing efficiency using the *APT* as a reporter gene. We show that the near PAMless SpRYCas9i effectively recognizes specific PAMs in *P. patens* that are not or poorly recognized by the native SpCas9. Pattern of mutations found using the SpRYCas9i are similar to the ones found with the SpCas9 and we could not detect off-target activity for the sgRNAs tested in this study. These findings contribute to advancing versatile genome editing techniques in plants.

**Supplementary Information:**

The online version contains supplementary material available at 10.1007/s11248-024-00381-1.

## Introduction

Genome editing is the deliberate and targeted manipulation of genetic materials in order to alter their information. Ever since the CRISPR (clustered regularly interspaced short palindromic repeat)/Cas system was reported, continuous improvements have been made allowing highly targeted editing of the genomes for basic and applied scientific research in many species, including plants (Hassan et al. 2021). However, limitations exist, one of them being due to the necessity for the CRISPR-Cas immune systems to distinguish self from nonself. For this, the Cas nucleases require a specific protospacer-adjacent motif (PAM) close to the target to be active. This requirement for Cas nucleases to recognize a specific PAM restrains the application of this technology for genome editing. For the most widely used SpCas9, this PAM corresponds to NGG, with a low tolerance for NAG and NGA PAMs. Several groups have worked to decrease or even remove the PAM restriction of Cas9 and highly flexible PAM spCas9, such as SpCas9-NG, xCas9, XNG-Cas9, or SpRY, have been used in crop plants like rice and tomato, and in one model plant, Arabidopsis (see Hassan et al. 2021 for a review). Notably, a SpRY variant was designed to have a highly relaxed PAM requirement (Walton et al. 2020). This near-PAMless variant of the Cas9 allowed for the generation of previously unattainable genetic variations in fungi and animals, but also in the plants Arabidopsis and rice (Li et al. 2021; Pan et al. 2021; Ren et al. 2021; Xu et al. 2021; Wu et al. 2022).

These near-PAMless Cas9 nucleases have increased significantly the number of sites that could be targeted, however, in general, efficiency of these variants does not match the efficiency of the original SpCas9 with a canonical NGG PAM. Various studies aimed at increasing efficiency of the Cas9 nucleases by modifying different structural parameters, such as the number of nuclear localization signals, the codon usage and the presence of introns in the coding sequence of the *Cas9* gene (Hassan et al. 2021). In particular, a study showed that the addition of Arabidopsis introns in the sequence of the coding sequence of a *Zea mays* codon optimized Cas9 improved significantly the editing efficiency in Arabidopsis and other dicotyledons (Grützner et al. 2021). The same study also showed that the presence of two NLS in the construct allowed for a better nuclear import, thus a better mutagenetic activity of the nuclease.

Because efficiency of editing for a given PAM by these near-PAMless Cas9 can vary from one species to another (Hassan et al. 2021), we decided to evaluate the efficiency of editing via the SpRY variant in the model plant *Physcomitrium patens*. To optimize SpRY Cas9 activity, we incorporated six introns in the SpRY Cas9 coding sequence and added NLS sequences in both the N-terminal and the C-terminal of the nuclease. Our work reveals that SpRYCas9i has varying efficiency with different PAMs, showing higher recognition of certain PAMs and comparable mutation patterns to SpCas9. Notably, this SpRYCas9i variant favored NRN PAMs similar to what was observed in human cells and zebrafish. Finally, we show that the SpRYCas9i’s off-target activity was minimal in *Physcomitrium patens*.

## Materials and methods

### Plant material and culture

We used the *Physcomitrium patens* ecotype Gransden pedigree Versailles (Haas et al. 2020) in this study. Tissue was routinely maintained and propagated on PpNH4 medium either by tissue picking or through tissue blending in sterile purified water (Schaefer et al. 1991). Culture chamber conditions were set at 60% humidity, temperature at 22 °C with a long day light cycle of 16 h of light (quantum irradiance of 80 μmol m^−2^ s^−1^) and 8 h of dark.

### Vector design and assembly

The pUbi_SpRYCas9i plasmid was assembled by GoldenBraid cloning (Sarrion-Perdigones et al. 2011) using a level 1 Alpha plant expression vector (pDGB3_alpha1, Plasmid #68,228 from Addgene) and the three modules, pZmUbi, SpRYCas9i and tRbcSE9. Module pZmUbi, was domesticated from plasmid pIPKb002 (Himmelbach et al. 2007), it derives from the *Zea mays* ubiquitin-1 promoter (GenBank: S94464). Module SpRYCas9i was synthesized (TwistBioscience), it derives from the zCas9i (Grützner et al. 2021) but contains 6 introns out of the 13 introns of the zCas9i and the 11 mutations of the SpRY Cas9 variant (Walton et al. 2020). Module tRbcSE9 was synthesized (TwistBioscience), it corresponds to the *Pisum sativum* ribulose bisphosphate carboxylase small subunits terminator (GenBank: OK586892). The control plasmid, pUbi_SpCas9, expressing the native Cas9, is described in Perroud et al. 2023. Maps of the plasmids are shown in Figures S1 and S2, respectively.

Guide RNA (sgRNA) sequences (Table S1) targeting the *APT* (Pp3c8_16590) gene were chosen using the webtool CRISPOR (Concordet and Haeussler 2018). Expression cassettes of the different sgRNAs, comprising the promoter of the *P. patens* U6 snRNA (Collonnier et al. 2017), the 5′-G-N(19)-3′ guide sequences targeting the *APT* gene and the tracrRNA scaffold, were synthesized by Twist Bioscience and subcloned into the pDONR207 vector by GatewayTM BP reaction (Invitrogen).

### Moss transfection and selection procedures

Moss protoplast isolation was performed from six day-old blended protonemal tissue as previously described (Charlot et al. 2022). 180 000 protoplasts were transfected with a total amount of 10 µg of circular plasmid DNA per transfection. After transfection, the dilution of the transfection reaction, its embedding in alginate and spreading on cellophane disks laid atop of PpNH4 medium supplemented with 0.33 M Mannitol were performed as previously described (Charlot et al. 2022). Plants on cellophane disks were then selected on PpNH4 supplemented with 10 μM 2-FA (Fluorochem) to select clones that were mutated at the *APT* locus (Collonnier et al. 2017). Growing plants after ten days were counted and individually sub-cultured on fresh PpNH4 medium until harvesting for genotyping.

### PCR and sequence analysis of the edited plants

Moss genomic DNA samples were isolated from 50 mg of fresh tissue in 96-well microtube plates as previously described (Lopez-Obando et al. 2016). Molecular analysis of the mutations induced in the *APT* gene was based on Sanger sequencing (Genoscreen, Lille, France) of PCR fragments using primers (Table S2) flanking the targeted *APT* locus. Predicted potential off-target loci (Table S3) were identified using the webtool CRISPOR and their molecular analysis was based on Sanger sequencing of PCR fragments using primers (Table S2) surrounding the identified loci.

### Graphics and statistical analysis

Graphs were generated with Excel. The Shapiro–Wilk test has been performed to evaluate replicate normality. The Levene’s test was used to evaluate variance homogeneity across the experiment. Subsequently, a paired t-test was performed to compare the sgRNA guide efficiency. All tests were performed in R studio (R 4.2.1) (Table S4, S5 and S6). Samples with mutation rate equal to 0 were excluded from the statistical analysis.

## Results and discussion

**Fig. 1:**
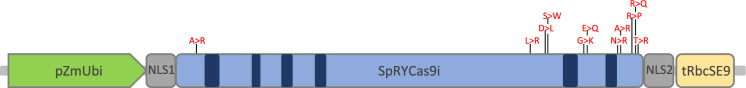
Structure of the cassette expressing the codon optimized SpRYCas9i gene. The expression cassette used for PEG-mediated transfection is schematically depicted. pZmUbi: *Zea mays* Ubiquitin promoter; SpRYCas9i: *Zea mays* codon-optimized Cas9 from *Streptococcus pyogenes* with 6 introns (dark blue boxes); tRbcSE9: *Pisum sativum* ribulose bisphosphate carboxylase small subunits terminator. Substitutions A61R/L1095R/D1135L/S1136W/G1218Q/E1219Q/N1317R/A1322R/R1333P/R1335Q/T1337R compared to the native SpCas9 (Q99ZW2) (from Walton et al. 2020) are indicated in red. (Color figure online)

Efficiency of genome editing in plants using the SpRY variant of the SpCas9 is generally lower compared to the original SpCas9 (Li et al. 2021; Ren et al. 2021). In order to improve the efficiency of SpRY we carried out optimization of its expression and developed a PAM-less Cas9 variant that we named SpRYCas9i. The SpRYCas9i gene contains the modifications described in Walton et al 2020, introns based on the zCas9i (Grützner et al. 2021) and two NLS targeting signals, a SV40 and a nucleoplasmin NLS sequences (Fig. 1).

**Fig. 2:**
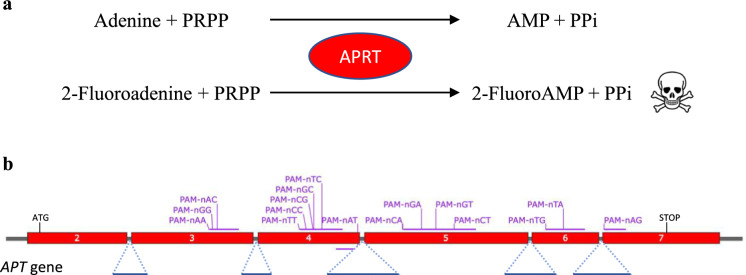
Schematic description of the *APT* reporter gene and positions of the sgRNAs. **a** The *APT* gene encodes the Adenine phosphoribosyltransferase (APRT) catalyses a phosphoribosyl transfer from Phosphoribosyl Pyrophosphate (PRPP) to adenine, forming adenosine monophosphate (AMP) and releasing pyrophosphate (PPi). In the presence of 2-Fluoroadenine APRT will form 2-FluoroAMP, a toxic compound for the cell. Cells where the *APT* gene has been knocked-out will survive on 2-Fluoroadenine.** b** Positions of the sgRNAs on the *APT* gene. Red boxes correspond to exons, blue lines to introns. The 16 sgRNAs positions are indicated in purple, at the top for sgRNAs that target forward strand, and at the bottom for sgRNA that target reverse strand. (Color figure online)

**Fig. 3:**
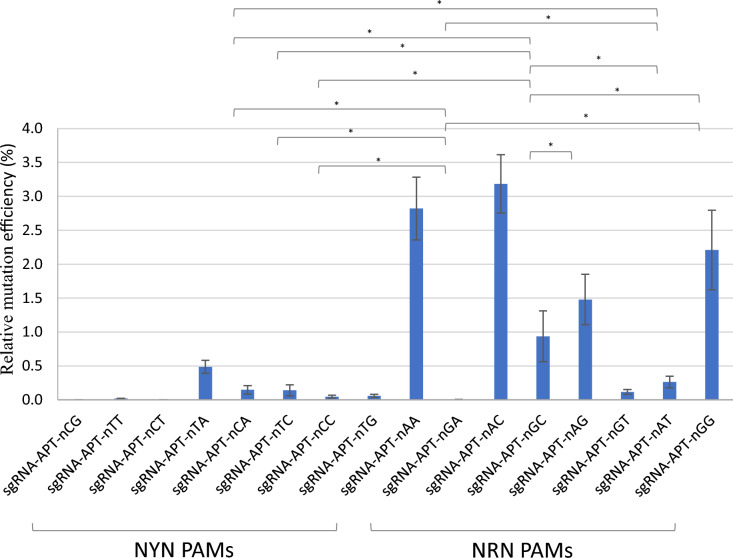
Editing of the *APT* reporter gene at NYN and NRN PAM targets. Mutation efficiency of the *APT* (*Adenine phosphoribosyltransferase*) gene using guides targeting NYN and NRN PAM (protospacer adjacent motif) targets, with SpRYCas9i. Relative mutation efficiency expresses the frequency of clones resistant to 2-Fluoroadenine among the population of regenerants. Error bars indicate ± SD (standard deviation) based on three independent experiments. Significant differences observed for sgRNA efficiencies paired comparison using paired t-test are marked. *(*p*-value ≤ 0.05).

To assess the edition efficiency of SpRYCas9i, we used the *APT* gene as a reporter of the genome editing. In short, the *APT* gene codes for the APRT enzyme, a Type I phosphoribosyltransferase family involved in the conversion process of adenine to adenosine monophosphate (AMP) in normal conditions. This APRT enzyme also has the capacity to convert adenine analogues such as the 2-Fluoroadenine (2-FA) into 2-Fluoro-AMP, a lethal compound for plants (Fig. 2a). This property can be used for counter-selection of the non-edited individuals as they will not be able to survive on a 2-FA-containing medium. To test the editing efficiency of SpRYCas9i for different PAMs, we designed 16 gRNAs targeting the *APT* gene with PAMs containing all possible nucleotide combinations in the second and third position (Fig. 2b). *P. patens* wild-type protoplasts were co-transfected by PEG-mediated transformation with two plasmids, one bearing the SpRYCas9i gene under the control of the maize Ubi promoter (pUbi_SpRYCas9i, Fig. 1, Fig. S1) and another bearing one of the 16 sgRNAs under the control of a *P. patens* U6 promoter. As a control, we used the native SpCas9 under the control of the maize Ubi promoter (pUbi_SpCas9, Fig. S2) with the same 16 sgRNAs. The relative mutation rates (expressed in percentages), using the different sgRNAs, were estimated by dividing the number of 2-FA-resistant plants by the number of regenerating plants observed just before the transfer on 2-FA. Mutation rate using the native SpCas9 and a sgRNA targeting a NGG PAM is close to 13% (Table 1). As expected, mutation rate of sgRNAs using a non NGG PAM is very low or null, with only one sgRNA, using a nAG PAM, leading to an important number of *apt* mutants (Table 1). Like the SpCas9, SpRYCas9i is recognizing the nGG PAM, but less efficiently (sixfold less) than the native Cas9 (Fig. 3, Table 2). The PAMs for which the efficiency of SpRYCas9i is the highest are NAA and NAC, which are not recognized at all by the SpCas9 (Table 1). In general, NRN PAMs are recognized significantly more efficiently by the SpRYCas9i compared to NYN PAMs (Fig. 3 and Table S5 for* p*-value of paired t-test, *p*-value ≤ 0.05 considered as significant). Interestingly, the sgRNA target APT locus containing the PAM that corresponds to the three first base pairs of the RNA scaffold, GTT, is not or very weakly mutated by the SpRYCas9i in *P. patens*. This is probably due to the detrimental self-target effect on the sgRNA sequence that could be observed with SpRY and SpCas9-NG, in rice (Qin et al. 2020; Xu et al. 2021).

**Table 1:** Mutation rates of the *APT* gene for different PAM using the native Cas9

PAM	Experiment						Average RME^b^ (%)
	#1			#2			
	Nb of reg	2-FA^R^ plants	RME^a^(%)	Nb of reg	2-FA^R^ plants	RME^a^(%)	
nCG	22,905	0	0,0	16,881	0	0,0	0.0
nTT	20,059	0	0,0	16,550	1	0,0	0.0
nCT	15,027	0	0,0	15,491	0	0,0	0.0
nTA	27,142	0	0,0	30,121	0	0,0	0.0
nCA	27,208	0	0,0	22,839	0	0,0	0.0
nTC	24,097	0	0,0	26,017	0	0,0	0.0
nCC	23,302	0	0,0	21,714	0	0,0	0.0
nTG	23,302	3	0,0	18,999	0	0,0	0.0
nAA	32,306	1	0,0	23,567	2	0,0	0.0
nGA	26,017	0	0,0	16,550	0	0,0	0.0
nAC	24,891	0	0,0	17,675	0	0,0	0.0
nGC	28,665	3	0,0	23,369	3	0,0	0.0
nAG	25,288	558	2,2	24,163	571	2,4	2.3
nGT	23,170	0	0,0	26,348	0	0,0	0.0
nAT	26,745	0	0,0	21,846	0	0,0	0.0
nGG	24,494	2988	12,2	21,052	2988	14,2	13.2

^a^Relative mutation efficiency expresses the frequency of 2-FA resistant clones among the population of regenerants

^b^Average was determined from the two independent experiments

**Table 2:** Mutation rates of the *APT* gene for different PAM using the SpRYCas9i variant

PAM	Experiment									Average RME^b^ (%)
	#1			#2			#3			
	Nb of reg	2-FA^R^ plants	RME^a^ (%)	Nb of reg	2-FA^R^ plants	RME^a^ (%)	Nb of reg	2-FA^R^ plants	RME^a^ (%)	
nCG	13,306	0	0.0	41,872	1	0.0	14,299	0	0.0	0.0 ± 0^b^
nTT	17,477	3	0.0	45,885	12	0.0	15,822	3	0.0	0.0 ± 0
nCT	10,393	0	0.0	43,858	0	0.0	20,191	0	0.0	0.0 ± 0
nTA	13,240	86	0.6	40,175	215	0.5	24,097	67	0.3	0.5 ± 0.1
nCA	11,585	30	0.3	27,845	48	0.2	17,940	2	0.0	0.1 ± 0.1
nTC	15,292	49	0.3	52,133	0	0.0	16,285	17	0.1	0.1 ± 0.1
nCC	14,961	13	0.1	47,940	23	0.0	22,508	1	0.0	0.0 ± 0.1
nTG	20,919	14	0.1	46,381	45	0.1	23,104	3	0.0	0.1 ± 0.1
nAA	16,087	567	3.5	29,004	918	3.2	19,595	347	1.8	2.8 ± 0.2
nGA	10,195	0	0.0	36,907	1	0.0	20,522	2	0.0	0.0 ± 0
nAC	12,313	445	3.6	36,369	1361	3.7	19,661	431	2.2	3.2 ± 0.7
nGC	11,122	75	0.7	33,721	601	1.8	21,250	75	0.4	0.9 ± 0.6
nAG	16,749	385	2.3	29,459	378	1.3	15,623	134	0.9	1.5 ± 0.5
nGT	14,101	24	0.2	36,451	53	0.1	24,031	9	0.0	0.1 ± 0.1
nAT	13,505	38	0.3	35,458	151	0.4	19,198	16	0.1	0.3 ± 0.1
nGG	12,247	315	2.6	33,017	1043	3.2	20,743	186	0.9	2.2 ± 0.9

^a^Relative mutation efficiency expresses the frequency of 2-FA resistant clones among the population of regenerants

^b^Average and standard deviations (± SD) were determined from the three independent experiments

In order to characterize the mutations generated by the SpRYCas9i, we amplified by PCR and sequenced the *APT* gene in 62 independent mutant plants obtained with the different sgRNAs (Fig. S3). As expected, all the mutations were located in the vicinity of the PAM targets of the SpRYCas9i-induced cleavage site and generated loss of APT function. These mutations consisted mainly of deletions and a few insertions, both with or without substitutions. Interestingly, for a majority of the deletions (53%), microhomologies (of 2–4 bp) could be detected between the end of the deletion itself and the sequence located just upstream of the deletion (Fig. S3). These different patterns of mutations are very similar to the ones already observed with the SpCas9 in *P. patens* (Collonnier et al. 2017), with a strong part of the deletions that could be explained by alt-EJ-mediated repair based on microhomologies (Oh and Myung 2022).

The relaxed PAM tolerances of near-PAMless Cas9 variants can, in principle, lead to recognition of additional off-target sites in the genome and SpRY has been shown to exhibit increased off-targeting compared to the native SpCas9 that could be decreased by using an HF1 version of SpRY (Walton et al. 2020). In order to evaluate the off-target activity of the SpRYCas9i in *P. patens*, off-target candidate loci (Table S3) were amplified and sequenced in 10 *apt* mutant plants generated with one sgRNA targeting a NRN type PAM (13 potential off-targets) and 10 *apt* mutant plants generated with one sgRNA targeting a NYN type PAM (9 potential off-targets). No mutation could be detected in the potential off-target sequences for any of the 20 tested plants.

We can conclude from these data that the structurally engineered SpRY is capable of recognizing NRN and several NYN PAMs in *P. patens*, but with a reduced efficiency with the latter. Mutations produced by SpRYCas9i are of the same nature as the ones produced by the SpCas9. The preference of SpRYCas9i for NRN PAMs mirrors what has been already observed in human cells, zebrafish, and rice (Walton et al. 2020; Xu et al. 2021; Liang et al. 2022). The near-PAMless variants have already been adapted into base editors and prime editor and should be adaptable to base editors and prime editors used in *P. patens* (Perroud et al. 2021; Guyon-Debast et al. 2021).

### Supplementary Information

Below is the link to the electronic supplementary material.

Supplementary file1 (PPTX 566 KB)

Supplementary file2 (DOCX 24 KB)
