# Supplementary material for: The SpRY Cas9 variant release the PAM sequence constraint for genome editing in the model plant Physcomitrium patens
Source: Transgenic Res. 2024 Apr 4;33(1-2):67–74. doi: 10.1007/s11248-024-00381-1 (PMC11021247; doi:10.1007/s11248-024-00381-1)
Supplement: Supplementary file 1 — Supplementary file1 (PPTX 566 KB) [file 11248_2024_381_MOESM1_ESM.pptx]

## Slide 1
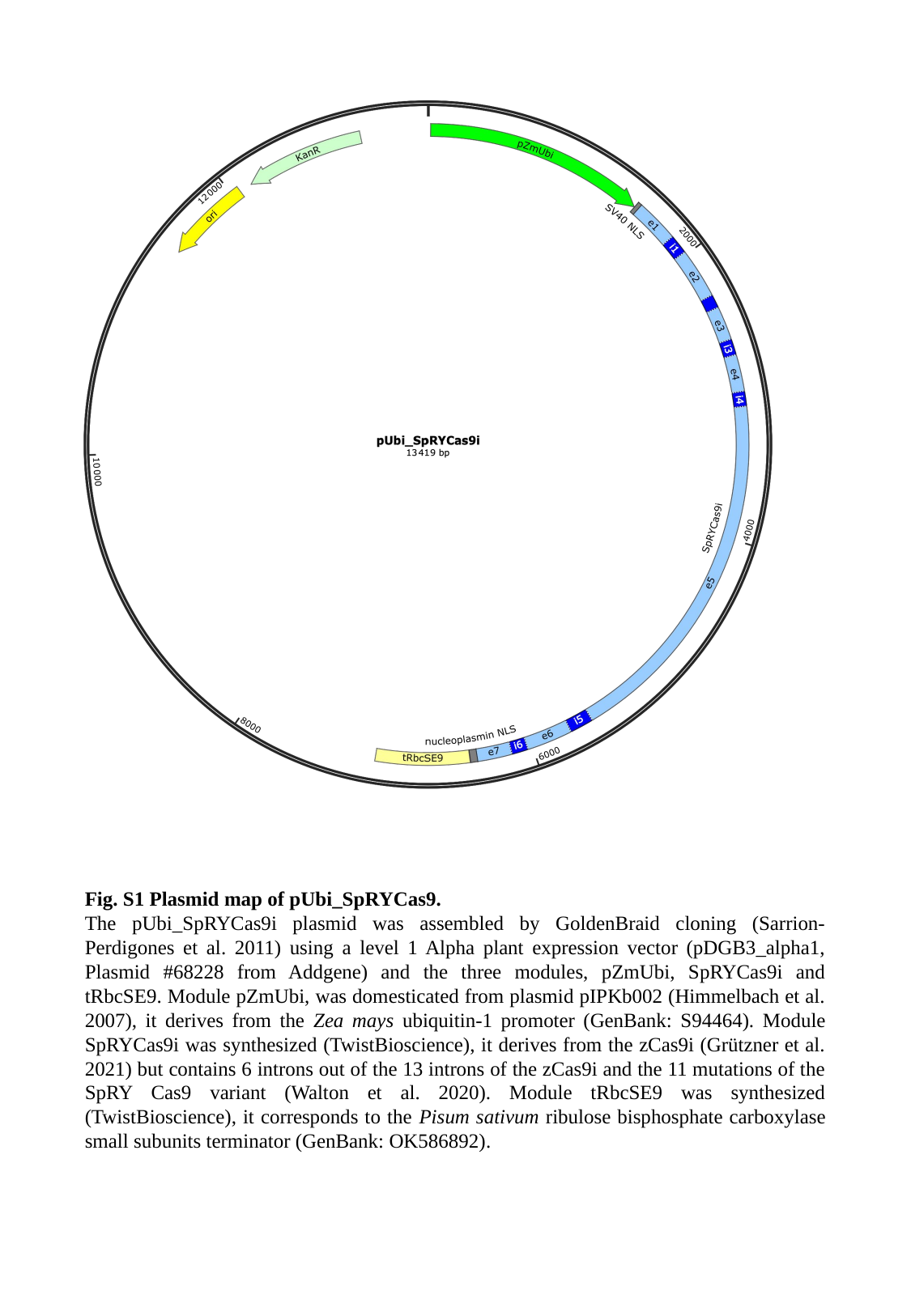

﻿Fig. S1 Plasmid map of pUbi_SpRYCas9.
The pUbi_SpRYCas9i plasmid was assembled by GoldenBraid cloning (Sarrion-Perdigones et al. 2011) using a level 1 Alpha plant expression vector (pDGB3_alpha1, Plasmid #68228 from Addgene) and the three modules, pZmUbi, SpRYCas9i and tRbcSE9. Module pZmUbi, was domesticated from plasmid pIPKb002 (Himmelbach et al. 2007), it derives from the Zea mays ubiquitin-1 promoter (GenBank: S94464). Module SpRYCas9i was synthesized (TwistBioscience), it derives from the zCas9i (Grützner et al. 2021) but contains 6 introns out of the 13 introns of the zCas9i and the 11 mutations of the SpRY Cas9 variant (Walton et al. 2020). Module tRbcSE9 was synthesized (TwistBioscience), it corresponds to the Pisum sativum ribulose bisphosphate carboxylase small subunits terminator (GenBank: OK586892).

## Slide 2
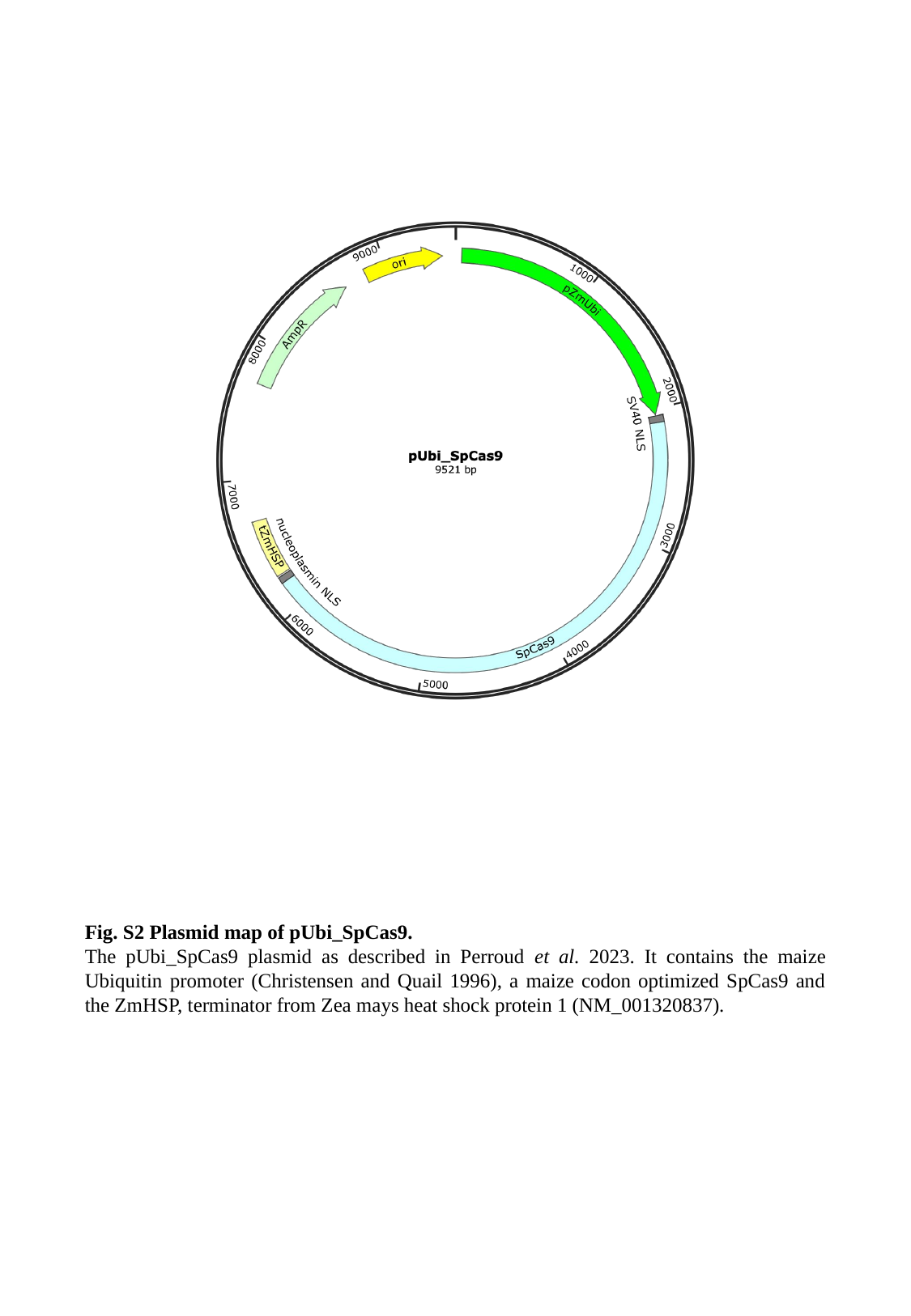

﻿Fig. S2 Plasmid map of pUbi_SpCas9.
The pUbi_SpCas9 plasmid as described in Perroud et al. 2023. It contains the maize Ubiquitin promoter (Christensen and Quail 1996), a maize codon optimized SpCas9 and the ZmHSP, terminator from Zea mays heat shock protein 1 (NM_001320837).

## Slide 3
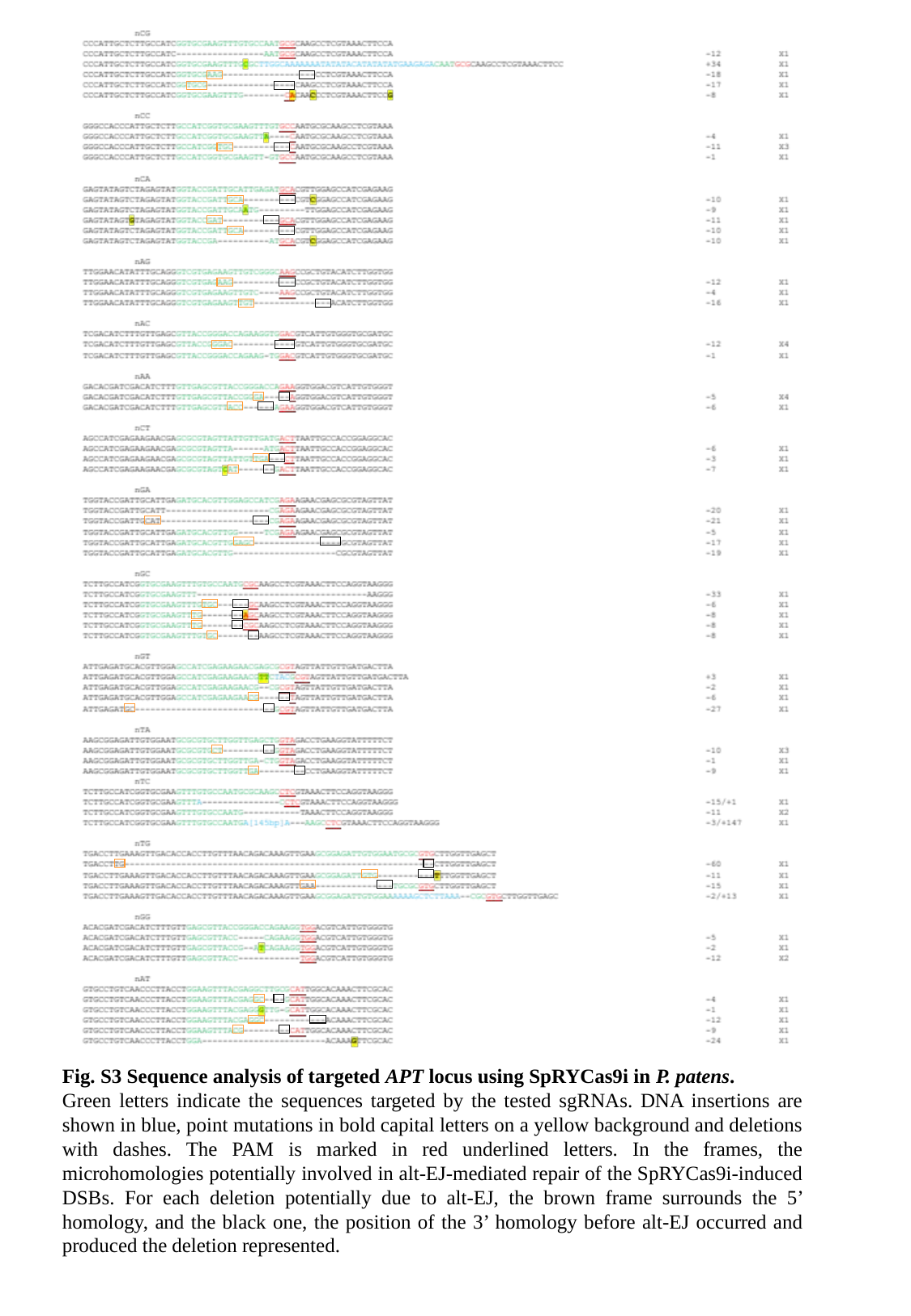

﻿Fig. S3 Sequence analysis of targeted APT locus using SpRYCas9i in P. patens.
Green letters indicate the sequences targeted by the tested sgRNAs. DNA insertions are shown in blue, point mutations in bold capital letters on a yellow background and deletions with dashes. The PAM is marked in red underlined letters. In the frames, the microhomologies potentially involved in alt-EJ-mediated repair of the SpRYCas9i-induced DSBs. For each deletion potentially due to alt-EJ, the brown frame surrounds the 5’ homology, and the black one, the position of the 3’ homology before alt-EJ occurred and produced the deletion represented.
