# Supplementary material for: The SpRY Cas9 variant release the PAM sequence constraint for genome editing in the model plant Physcomitrium patens
Source: Transgenic Res. 2024 Apr 4;33(1-2):67–74. doi: 10.1007/s11248-024-00381-1 (PMC11021247; doi:10.1007/s11248-024-00381-1)
Supplement: Supplementary file 2 — Supplementary file2 (DOCX 24 KB) [file 11248_2024_381_MOESM2_ESM.docx]

**Table S1: sgRNA used in this study.**

| **Name** | **Target sequence 5’ - 3’** | **PAM** |
| --- | --- | --- |
| PAM-nAA | GTTGAGCGTTACCGGGACCA | GAA |
| PAM-nAT | GGAAGTTTACGAGGCTTGCG | CAT |
| PAM-nAC | GTTACCGGGACCAGAAGGTG | GAC |
| PAM-nAG | GTCGTGAGAAGTTGTCGGGC | AAG |
| PAM-nTT | GCTCTTGCCATCGGTGCGAA | GTT |
| PAM-nTA | GCGCGTGCTTGGTTGAGCTG | GTA |
| PAM-nTC | GTTTGTGCCAATGCGCAAGC | CTC |
| PAM-nTG | GCGGAGATTGTGGAATGCGC | GTG |
| PAM-nCC | GCCATCGGTGCGAAGTTTGT | GCC |
| PAM-nCA | GGTACCGATTGCATTGAGAT | GCA |
| PAM-nCT | GCGCGTAGTTATTGTTGATG | ACT |
| PAM-nCG | GGTGCGAAGTTTGTGCCAAT | GCG |
| PAM-nGG | GAGCGTTACCGGGACCAGA | AGG |
| PAM-nGA | GATGCACGTTGGAGCCATCG | AGA |
| PAM-nGT | GCCATCGAGAAGAACGAGCG | CGT |
| PAM-nGC | GTGCGAAGTTTGTGCCAATG | CGC |

**Table S2:** **Primers used in this study.**

| **Name** | **Sequence 5’-3’** |
| --- | --- |
| PpAPT 4 | CTGTTGCAATGCAGGAGTAGGTGATGC |
| PpAPT 14 | AGATGTCGGCCTCCAAGGATG |
| PpAPT 5 | ACAAGGTGGTGTCAACTTTCAAGG |
| PpAPT 15 | TATGTTCCGAGATGTGACGAC |
| PpAPT 44 | AGGATTGGCACTTCATAGAAC |
| offtg NTG fw 1 | GCTCTTGTCTCTAAAACTGCTGTG |
| offtg NTG fw 2 | CCAACTCACTGTGCCTCGAA |
| offtg NTG fw 3 | CCACAGGAATTCAATGGCCG |
| offtg NTG fw 4 | CGGACTCCTGCAGATGACTC |
| offtg NTG fw 5 | CCCGGTGGCATTGATACACA |
| offtg NTG fw 6 | CCCTCATCCTGACAAACGGT |
| offtg NTG fw 7 | GTGTGCGCATCACTTCAACA |
| offtg NTG fw 8 | ATTGAGCTTCTCTCGCCGTT |
| offtg NTG fw 9 | CCTAGGAGCCAGGCAAACAA |
| offtg NTG fw 10 | GGGACATGGGATTGATTGCC |
| offtg NTG rv 1 | CCCCGTTAGCCGTCAAACAT |
| offtg NTG rv 2 | TCCTTCATCAGCACACACCC |
| offtg NTG rv 3 | GAGTGTGAATGCTCAAGCCG |
| offtg NTG rv 4 | TCACGTGTGGGGTAACATGG |
| offtg NTG rv 5 | CAGTGACGTTCCGGTTAGCA |
| offtg NTG rv 6 | TGAAATGTGAGTTGTGAAATGGCT |
| offtg NTG rv 7 | CCCCTACAGTGCTGCTTTCT |
| offtg NTG rv 8 | ATGTAGGGAAACTCCCGCAA |
| offtg NTG rv 9 | AGCTTAAAATTCAAGATTGCCCCT |
| offtg NTG rv 10 | CTTGCGCGACTTCGTTTCTC |
| offtg NAT fw 1 | TCCGAAACCAAACCCCTTCC |
| offtg NAT fw 2 | GTCACACGAGGGCTTCTCTC |
| offtg NAT fw 3 | ACCCCAATGCGTAGCCAATA |
| offtg NAT fw 4 | TGTCGTCGCAGAACATGGAA |
| offtg NAT fw 5 | ATTCTGGGAGGATGTGGGGA |
| offtg NAT fw 6 | TCATACAAGCAGCAGTGCCA |
| offtg NAT fw 7 | GTTGACAGCATCAATGAGACCT |
| offtg NAT fw 8 | GTGGCTATACTAGTGCCAAAAGA |
| offtg NAT fw 9 | AGTTGGAAGACACACCACAA |
| offtg NAT rv 1 | TCCCCTTCTCCCTTCTTCGT |
| offtg NAT rv 2 | CTCGTGCCAAATCATGGAGC |
| offtg NAT rv 3 | AGAGCCGTGAAACCCTAAGC |
| offtg NAT rv 4 | TCTTCGTCATGCAGTTCGCT |
| offtg NAT rv 5 | GCGATCGGATAACAATGCGG |
| offtg NAT rv 6 | TCACCATCGCCTTCAGCAAT |
| offtg NAT rv 7 | AGAGGTTGGGTAGCATACAACA |
| offtg NAT rv 8 | GCTCCTGCACCCAAGAGAAT |
| offtg NAT rv 9 | GCATGTACACATGGGTTTCCA |

**Table S3: Sequences and positions of possible off target sites for two sgRNAs targeting the *APT* gene.** **Each off-target regions where sequenced (primers in Table S5) in 10 independent plants mutated at the respective on-target loci (*APT* gene).**

|  | Target region | Locus on *P. patens* genome | RME (%) |
| --- | --- | --- | --- |
| On target | GCGGAGATTGTGGAATGCGCGTG | Ch08: 10811739-10811717 | 0,1% |
| Off-target 1 | GCAGAGATTGTGGAACGAGCACG | Ch10: 15152355-15152377 | 0,00% |
| Off-target 2 | GAGGAGATGGTGGAATGCGATGA | Ch25: 3874952-3874974 | 0,00% |
| Off-target 3 | TCGGAGATTGTGGAATCTGCCGA | Ch01: 16687793-16687815 | 0,00% |
| Off-target 4 | GCTAAGCTTGTGGAATGCGCCGT | Ch18: 12805537-12805559 | 0,00% |
| Off-target 5 | GCGGAGATTGTGGCAGGCGAGGA | Ch04: 16375778-16375800 | 0,00% |
| Off-target 6 | GCGGAGATTGTGGAACGCAGCGA | Ch04: 6933129-6933151 | 0,00% |
| Off-target 7 | GCGGAGAATGTGCCATGCGCTGC | Ch02: 6718657-6718679 | 0,00% |
| Off-target 8 | GCGGAGGTTGTGGAGTGCGGTGT | Ch01: 16575823-16575845 | 0,00% |
| Off-target 9 | GCGGAGATTGTGGCATACGTGAC | Ch23: 5670603-5670625 | 0,00% |
| Off-target 10 | ACGGAGATTGTGGAATTGGCTTC | Ch13: 8358116-8358138 | 0,00% |
| Off-target 11 | ACGAAGATTGTGGAGTGCGCGAA | Ch13: 5719059-5719081 | 0,00% |
| Off-target 12 | GTGGAGATTGTGCAATGTGCCCC | Ch07: 15234304-15234326 | 0,00% |
| Off-target 13 | GTGGAGATTGTGGAATGTGCTTT | Ch09: 37792-37814 | 0,00% |

|  | Target region | Locus on *P. patens* genome | RME (%) |
| --- | --- | --- | --- |
| On target | GGAAGTTTACGAGGCTTGCGCAT | Ch08: 10812424-10812446 | 0,3% |
| Off-target 1 | GGAAGTATCCGAGGCTTGAGCAG | Ch01: 4690002-4690024 | 0,00% |
| Off-target 2 | GCCAGTTTACGAGCCTTGCGCAG | Ch13: 9325280-9325302 | 0,00% |
| Off-target 3 | GTCAGTTTAAGAGGCTTGCGCCG | Ch01: 25732895-25732917 | 0,00% |
| Off-target 4 | GGAAGTTTACGAGTCTTTCTGAA | Ch23: 5205073-5205095 | 0,00% |
| Off-target 5 | GGACGTTTTCGATGCTTGCGTTC | Ch21: 5441816-5441838 | 0,00% |
| Off-target 6 | TGAAGTTTACGAAGCTTGTGACC | Ch22: 5667166-5667188 | 0,00% |
| Off-target 7 | GGAAGTTTACAAGGCTTACAATA | Ch03: 1476782-1476804 | 0,00% |
| Off-target 8 | AGAAGTTTACAAGGCTTGCAACA | Ch12: 1397277-1397299 | 0,00% |
| Off-target 9 | GGAAGTTTACAAGGCTTGCAATA | Ch05: 13311632-13311654 | 0,00% |

**Table S4: Data analysis of Table 2 using the Shapiro-Wilt test.**

| sgRNA | p_Value |
| --- | --- |
| nTA | 0.6368868 |
| nCA | 0.6368868 |
| nTC | 0.6368868 |
| nCC | 0 |
| nTG | 0 |
| nAA | 0.3171779 |
| nAC | 0.1139327 |
| nGC | 0.3913844 |
| nAG | 0.5367371 |
| nGT | 1 |
| nAT | 0.6368868 |
| nGG | 0.485463 |

Shapiro-Wilk test: sgRNA nCC and nTG have a p-value lower than 0.05, they are probably not normally distributed, hence rejected for the t-test analysis.

**Table S5: Data analysis of Table 2 using the Levene's Test for Homogeneity of Variance (center = median)**

| **Df** | **F value** | **Pr(>F)** |
| --- | --- | --- |
| 11 | 0.9515 | 0.5122 |

Since the p-value (0.5122) is greater than the significance level of 0.05, we fail to reject the null hypothesis. Therefore, there is not enough evidence to conclude that the variances are significantly different across groups. Hence, we can assume homogeneity of variances.

**Table S6: Data analysis of Table S4 using the paired t-test.**

|  | nTA | nCA | nTC | nAA | nAC | nGC | nAG | nGT | nAT | nGG |
| --- | --- | --- | --- | --- | --- | --- | --- | --- | --- | --- |
| nTA | NA | 0.074 | 0.056 | 0.042 | 0.027 | 0.361 | 0.126 | 0.032 | 0.184 | 0.122 |
| nCA |  | NA | 0.802 | 0.033 | 0.022 | 0.197 | 0.079 | 0.567 | 0.468 | 0.093 |
| nTC |  |  | NA | 0.033 | 0.022 | 0.185 | 0.076 | 0.770 | 0.345 | 0.091 |
| nAA |  |  |  | NA | 0.665 | 0.053 | 0.121 | 0.033 | 0.036 | 0.529 |
| nAC |  |  |  |  | NA | 0.028 | 0.061 | 0.023 | 0.024 | 0.336 |
| nGC |  |  |  |  |  | NA | 0.421 | 0.177 | 0.239 | 0.207 |
| nAG |  |  |  |  |  |  | NA | 0.076 | 0.092 | 0.424 |
| nGT |  |  |  |  |  |  |  | NA | 0.200 | 0.089 |
| nAT |  |  |  |  |  |  |  |  | NA | 0.102 |
| nGG |  |  |  |  |  |  |  |  |  | NA |

In yellow: sgRNA paired comparison with a p-value<= 0.05, considered as significative
